# The effect of acid–base clustering and ions on the growth of atmospheric nano-particles

**DOI:** 10.1038/ncomms11594

**Published:** 2016-05-20

**Authors:** Katrianne Lehtipalo, Linda Rondo, Jenni Kontkanen, Siegfried Schobesberger, Tuija Jokinen, Nina Sarnela, Andreas Kürten, Sebastian Ehrhart, Alessandro Franchin, Tuomo Nieminen, Francesco Riccobono, Mikko Sipilä, Taina Yli-Juuti, Jonathan Duplissy, Alexey Adamov, Lars Ahlm, João Almeida, Antonio Amorim, Federico Bianchi, Martin Breitenlechner, Josef Dommen, Andrew J. Downard, Eimear M. Dunne, Richard C. Flagan, Roberto Guida, Jani Hakala, Armin Hansel, Werner Jud, Juha Kangasluoma, Veli-Matti Kerminen, Helmi Keskinen, Jaeseok Kim, Jasper Kirkby, Agnieszka Kupc, Oona Kupiainen-Määttä, Ari Laaksonen, Michael J. Lawler, Markus Leiminger, Serge Mathot, Tinja Olenius, Ismael K. Ortega, Antti Onnela, Tuukka Petäjä, Arnaud Praplan, Matti P. Rissanen, Taina Ruuskanen, Filipe D. Santos, Simon Schallhart, Ralf Schnitzhofer, Mario Simon, James N. Smith, Jasmin Tröstl, Georgios Tsagkogeorgas, António Tomé, Petri Vaattovaara, Hanna Vehkamäki, Aron E. Vrtala, Paul E. Wagner, Christina Williamson, Daniela Wimmer, Paul M. Winkler, Annele Virtanen, Neil M. Donahue, Kenneth S. Carslaw, Urs Baltensperger, Ilona Riipinen, Joachim Curtius, Douglas R. Worsnop, Markku Kulmala

**Affiliations:** 1 Department of Physics, University of Helsinki, PO Box 64, 00014 Helsinki, Finland; 2 Laboratory of Atmospheric Chemistry, Paul Scherrer Institute, 5232 Villigen, Switzerland; 3 Institute for Atmospheric and Environmental Sciences, Goethe-University of Frankfurt, Altenhöferallee 1, 60438 Frankfurt am Main, Germany; 4 CERN, 1211 Geneva, Switzerland; 5 Helsinki Institute of Physics, University of Helsinki, PO Box 64, 00014 Helsinki, Finland; 6 Department of Applied Physics, University of Eastern Finland, PO Box 1627, 70211 Kuopio, Finland; 7 Department of Environmental Science and Analytical Chemistry (ACES) & Bolin Centre for Climate Research, Stockholm University, 10691 Stockholm, Sweden; 8 SIM, University of Lisbon and University of Beira Interior, 1749-016 Lisbon, Portugal; 9 Institute for Atmospheric and Climate Science, ETH Zurich, 8092 Zurich, Switzerland; 10 Institute for Ion Physics and Applied Physics, Technikerstraße 25, 6020 Innsbruck, Austria; 11 Division of Chemistry and Chemical Engineering, California Institute of Technology, 1200 E. California Boulevard Pasadena, California 91125, USA; 12 School of Earth and Environment, University of Leeds, Leeds LS2 9JT, UK; 13 Finnish Meteorological Institute, Atmospheric Research Centre of Eastern Finland, PO Box 1627, 70211 Kuopio, Finland; 14 Ionicon Analytik GmbH, Eduard-Bodem-Gasse 3, 6020 Innsbruck, Austria; 15 University of Vienna, Faculty of Physics, Boltzmanngasse 5, 1090 Vienna, Austria; 16 Finnish Meteorological Institute, PO Box 501, 00101 Helsinki, Finland; 17 Department of Chemistry, University of California, Irvine, California, 92697 USA; 18 Leibniz Institute for Tropospheric Research, Permoserstrasse 15, 04318 Leipzig, Germany; 19 Center for Atmospheric Particle Studies, Carnegie Mellon University, Doherty Hall 2116, Pittsburgh, Pennsylvania 15213, USA; 20 Aerodyne Research Inc., Billerica, Massachusetts 01821-3976, USA

## Abstract

The growth of freshly formed aerosol particles can be the bottleneck in their survival to cloud condensation nuclei. It is therefore crucial to understand how particles grow in the atmosphere. Insufficient experimental data has impeded a profound understanding of nano-particle growth under atmospheric conditions. Here we study nano-particle growth in the CLOUD (Cosmics Leaving OUtdoors Droplets) chamber, starting from the formation of molecular clusters. We present measured growth rates at sub-3 nm sizes with different atmospherically relevant concentrations of sulphuric acid, water, ammonia and dimethylamine. We find that atmospheric ions and small acid-base clusters, which are not generally accounted for in the measurement of sulphuric acid vapour, can participate in the growth process, leading to enhanced growth rates. The availability of compounds capable of stabilizing sulphuric acid clusters governs the magnitude of these effects and thus the exact growth mechanism. We bring these observations into a coherent framework and discuss their significance in the atmosphere.

## Abstract (web summary)

The growth rates of freshly formed aerosol particles influence what fraction of these can reach sizes large enough to affect cloud formation and climate. Here, the authors show that the nano-particle growth in a sulphuric acid containing system can be enhanced by the presence of ions or small acid-base clusters.

## Graphical abstract

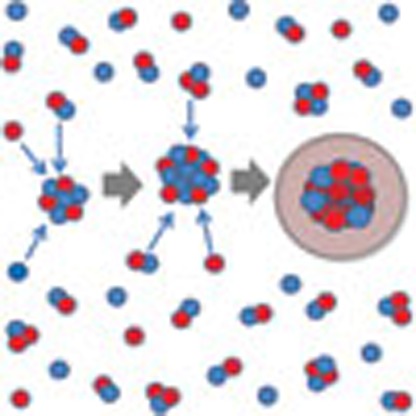

Atmospheric new-particle formation is a major source of cloud condensation nuclei (CCN)^1^ and a large contributor to the current uncertainty associated with aerosol–cloud–climate interactions. Significant effort in both the field and laboratory settings has been put into unravelling aerosol particle formation mechanisms and improving predictions and parameterizations of particle formation rates^2^,^3^,^4^,^5^. However, new-particle formation and subsequent CCN production is limited by the particle growth rate (GR) in the 1–3 nm diameter size range, rather than by the formation rate of molecular clusters, which are frequently present in the atmosphere^3^,^6^,^7^,^8^. As particles smaller than 3 nm contain only a handful of molecules, they diffuse almost as rapidly as gases and their survival probability depends critically on their growth rate. Interpretation of field measurement data suffers seriously from problems in isolating the different growth processes, such as vapour condensation, coagulation and various chemical reactions. The roles of different precursor vapours and ions in growth are still unclear, as the relevant concentrations in the atmosphere are extremely low. Laboratory studies conducted so far have not directly addressed the nano-particle growth issue.

Even though sulphuric acid vapour has been established as the main driving component for particle formation in the atmosphere^2^,^6^,^9^,^10^, numerous field studies have shown that the growth rates often substantially exceed the predictions based on measured sulphuric acid concentrations, and the remaining fraction of growth has usually been attributed to condensation of low-volatility organic vapours^3^,^8^,^10^,^11^,^12^,^13^. This is supported by quantitative measurements of the particle composition in the size range above 10 nm (refs 13, 14). It has recently been speculated that amines or other alkaline vapours could also participate in the growth process—possibly via salt formation^15^,^16^,^17^,^18^—and aminium salts have indeed been observed in 10 nm particles^15^. It has also been debated to what extent the electric charge of the initial clusters can assist the growth process, which affects the relative importance of the neutral and charged particle formation pathways^19^,^20^,^21^.

In this study, the observed nano-particle growth can be directly connected to the precursor vapour concentrations and the gas-to-particle formation processes starting from molecular clusters. Although sulphuric acid is responsible for the nano-particle growth in all the studied experimental conditions, the exact growth mechanism is strongly tied to the presence of compounds capable of stabilizing sulphuric acid clusters. At low concentrations of base compounds, nano-particles grow mainly via the uptake of sulphuric acid and base monomers according to the traditional theory for vapour condensation, and the growth of the smallest particles is accelerated by the presence of electric charges. However, when a strongly basic compound is present, the nano-particle growth can be greatly enhanced by acid-base clusters. To the best of our knowledge, this is the first time these phenomena have been observed and quantified in a controlled system, which is required for understanding their role in nature.

## Results

### The growth rate of sub-3 nm particles in different systems

We measured the particle diameter GR (usually expressed in nm h^−1^) under extremely well-controlled conditions in the CLOUD (Cosmics Leaving OUtdoors Droplets) chamber at CERN using multiple techniques and instruments to cover the size range from about 1 to 100 nm in particle diameter (see Methods)^2^. A set of experiments, referred here as binary experiments, were dedicated to studying particle formation in a system of sulphuric acid (H_2_SO_4_) and water vapour under different conditions, with other condensable compounds present only as impurities in the chamber. The ammonia concentration in these experiments was mostly below the detection limit (35 p.p.t.v. (parts per trillion volume) in CLOUD3 campaign and about 5 p.p.t.v. in CLOUD4 and CLOUD7)^22^,^23^. In separate experiments, ammonia (NH_3_; about 100–1,400 p.p.t.v.) or dimethylamine (DMA; about <5–70 p.p.t.v.) was added to the chamber to study ternary nucleation and the role of acid-base clustering and nano-particle growth. To capture atmospherically relevant conditions, the amine concentration was kept several orders of magnitude lower than the concentrations used in previous laboratory studies^16^,^18^. The CLOUD measurement sequence^2^ allowed us to compare purely neutral experiments, in which all ions were removed with a high-voltage clearing field, to experiments with ions present in the chamber, but otherwise identical conditions. The ions were created either by natural galactic cosmic rays or by enhancing the ionization with a pion beam from the CERN proton synchrotron.

**Figure 1:**
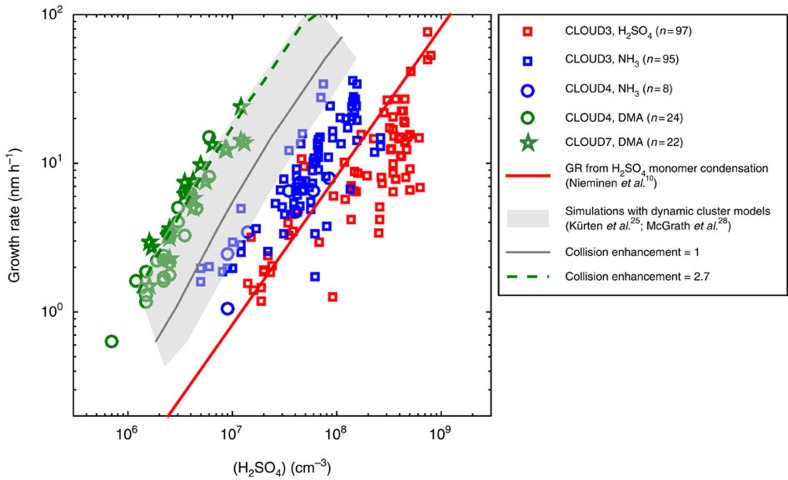
Growth rates in different systems. Growth rates of 2 nm particles determined with the appearance time method between 1.5 and 2.5 nm (ref. 24) as a function of the measured sulphuric acid (H_2_SO_4_) concentration with different amounts of ammonia (NH_3_) and dimethylamine (DMA) in the chamber. In the red data points, ammonia was present only as an impurity (<35 p.p.t.v. for CLOUD3 campaign, <5 p.p.t.v. for CLOUD4 and CLOUD7); for the blue points NH_3_ (100–1,400 p.p.t.v.) was added, and for green points DMA (5–70 p.p.t.v.) was added to the chamber. Squares represent experiments during the CLOUD3 campaign at varying temperatures (*T*=248–293 K) and relative humidities (RH=10–40%), while circles represent CLOUD4 and stars the CLOUD7 campaign, each at *T*=278 K, RH=38%. Sample size (*n*) for each system is given in the legend. The red line is the mass flux growth rate calculated from the sulphuric acid monomer concentration (at *T*=278 K)^10^, and the grey shaded area represents the appearance time growth rate determined from cluster population simulations^25^,^28^ assuming zero cluster evaporation rates and hard-sphere collision rates. A factor of 0.5–3 uncertainty in the collision rates (giving the limits of the shaded area) arises from the possibly non-unity sticking factors, uncertainty in the geometric cross-section of the clusters, and possible dipole–dipole enhancements in the collision rates. A collision enhancement factor of 2.7 (green dashed line) gives a good match between the simulated and measured data points in the sulphuric-acid–DMA system (see also Kürten *et al*.^25^).

We developed a method to analyse particle growth rates in the size range below 3 nm based on the appearance times of newly formed clusters^24^ (Supplementary Figs 1 and 2). The GRs of particles between about 1.5 and 2.5 nm in mobility diameter are presented in Fig. 1 as a function of the measured H_2_SO_4_ monomer concentration. The GRs varied almost linearly with the H_2_SO_4_ concentration at any given NH_3_ or DMA concentration. In the binary experiments, the measured GRs were equal or slightly lower than the GRs predicted by the mass flux of hydrated H_2_SO_4_ monomers on 2 nm particles^10^. However, it must be kept in mind that the appearance time growth rates are not exactly comparable to the mass flux growth rates especially in the sub-3 nm size range (see Methods). The addition of alkaline vapours to the system caused an increase in the GR at a given H_2_SO_4_ monomer concentration. The GR increased by a factor 2–3 with the addition of >100 p.p.t.v. NH_3_, and by an additional factor of ∼10 with the addition of >5 p.p.t.v. DMA. Further increase in the alkaline vapour concentrations did not yield a greater enhancement. The growth rates especially in the DMA system were much larger than could be expected based on the mass flux calculated from the measured H_2_SO_4_ monomer concentration^10^, even when accounting for co-condensation of bases. The GR measurements were verified by calculating them independently from different instruments using different measurement principles, so the effect of particle composition on the GR measurement could be excluded (Supplementary Fig. 3).

### The effect of acid–base clusters on growth

It has been shown that sulphuric acid rapidly forms clusters when DMA is present^4^,^25^,^26^. We identified neutral clusters consisting of up to 12 sulphuric acid and 14 amine molecules^25^ (Supplementary Fig. 4) using a nitrate chemical ionization time-of-flight mass spectrometer (CI-APi-TOF)^27^. The measured high cluster concentrations indicate that cluster evaporation is suppressed in the presence of DMA, and that clustering proceeds near to or at the kinetic limit^25^. Indeed, the growth rates determined from cluster population simulations^25^,^28^ (see Methods, and Supplementary Fig. 5) assuming low or zero evaporation rate were close to those observed for the experiments with added DMA (Fig. 1 shaded area). Earlier also McMurry^29^ has studied photochemical aerosol formation using a simplified theoretical model, which takes into account both monomer and cluster collisions and assumes evaporation to be negligible, and found good agreement between measured and modelled size distributions (in the size range above 10 nm), especially with enhanced cluster collision rates in the model.

**Figure 2:**
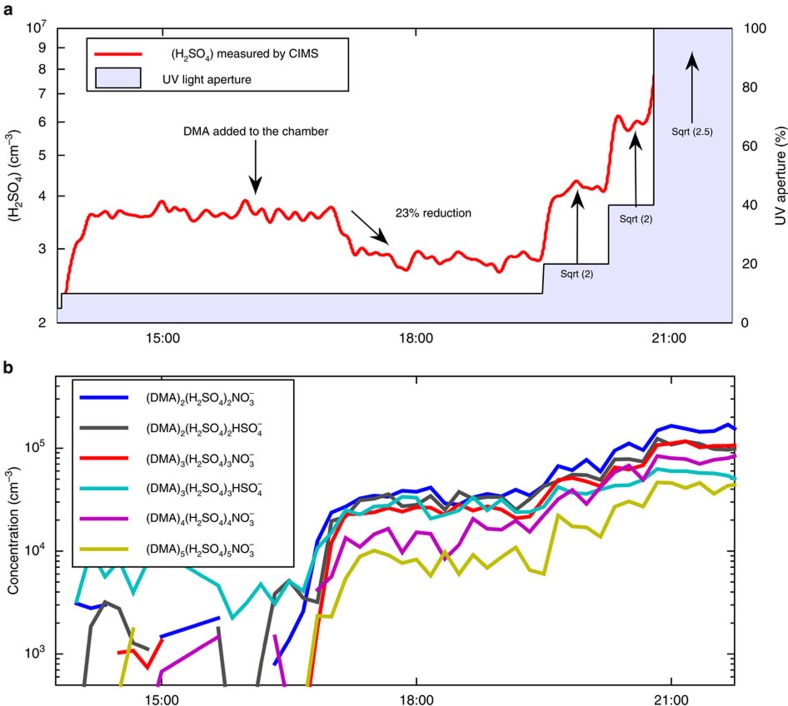
First addition of DMA. (**a**) Sulphuric acid monomer concentration (red line) measured by the CIMS as a function of time. In the beginning of the experiment (14:00–16:00) no dimethylamine was added to the chamber and the DMA concentration was below detection limit. After addition of DMA (the DMA flow was started around 16:00, after which it took some time to reach the chamber) the measured H_2_SO_4_ monomer concentration decreased, although the production rate of H_2_SO_4_ remained constant. After 19:30 the ultra violet light intensity (blue shaded area) was stepwise increased to increase production of sulphuric acid. (**b**) Concentrations of selected DMA–H_2_SO_4_ clusters measured with the CI-APi-TOF for the same time period.

The results imply that in the experiments with added DMA, a large portion of the sulphuric acid available for growth is bound to larger clusters, which are not measured by the chemical ionization mass spectrometer (CIMS) technique routinely used for sulphuric acid measurement, as it only includes the single molecules of H_2_SO_4_ (potentially clustered with water, ammonia or amine). To verify this, we designed an experiment in which DMA was added into a clean chamber while the H_2_SO_4_ production rate was kept constant. The signal of sulphuric acid monomer decreased shortly after the DMA was added (Fig. 2a), accompanied by a concurrent increase in the signal of larger clusters in the CI-APi-TOF (Fig. 2b). In binary experiments, the sulphuric acid concentration in the chamber depended almost linearly on the ultra violet light illumination, because the sulphuric acid concentration is controlled almost entirely by a balance between production (which is proportional to ultra violet) and deposition to the wall. However, this was not the case after DMA was added, as the measured sulphuric acid monomer concentration rose much less than expected when doubling the ultra violet intensity (Fig. 2a). This indicates that a large fraction of the photochemically produced sulphuric acid was accumulated in the larger clusters, and thus not detected by the CIMS measurement. The exact relation between the monomer and total sulphuric acid is discussed elsewhere^30^.

In the experiments with sulphuric acid and ammonia, the cluster formation rate was lower for a given acid concentration than with DMA^4^, and a larger fraction of sulphuric acid was available as free or hydrated monomers. Therefore the increase of the GR with respect to the binary case (Fig. 1) is likely to be mainly due to reduced evaporation rates (Supplementary Fig. 6) and/or contribution of ammonia molecules to the cluster size, as the growth is expected to progress mostly by consecutive additions of possibly hydrated sulphuric acid molecules and ammonia molecules, although cluster–cluster collisions may also have some contribution especially at high sulphuric acid concentrations.

### The effect of electric charges on growth

**Figure 3:**
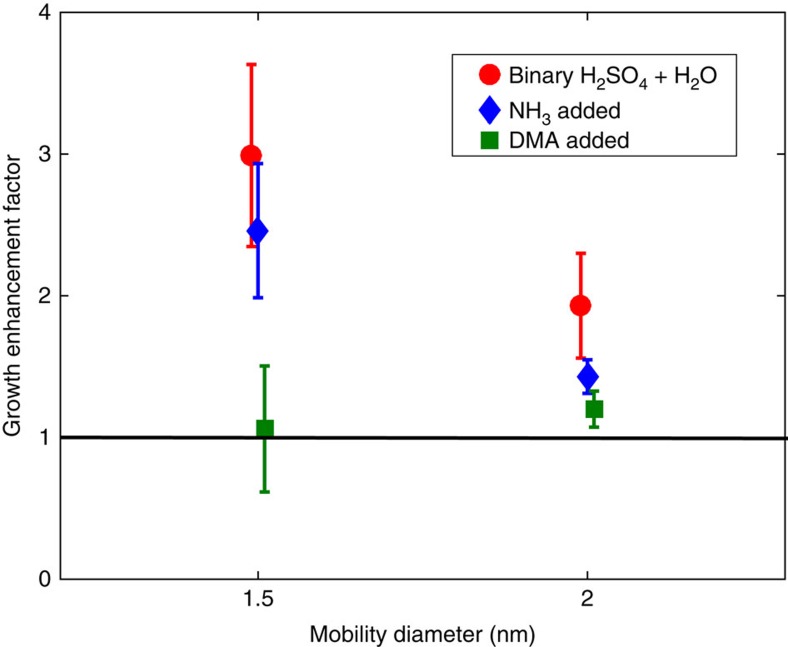
The effect of ions. The growth enhancement factor (GEF) due to the presence of ions in the chamber as a function of diameter in different systems. The GEF was determined as the ratio of the growth rate of the total particle population in a charged run and an identical neutral run. Each data point is at the mean value of all the experiments for which the GEF was possible to determine for the given system. Error bars give the s.e.m. The black line shows the case with no growth enhancement. Note that the magnitude of the enhancement is also dependent on the charged fraction of the particle population.

It has been speculated^19^,^20^,^21^ that electric charges on clusters can also enhance the growth rate of the aerosol population significantly, either due to increased condensation of polar vapours on the charged clusters (increased collision rate) or by making the clusters more stable (decreased evaporation rate). To date, however, the magnitude of this growth enhancement or its dependence on particle size and composition has not been experimentally verified. We compared the GR of particles in otherwise identical experiments but with or without ions present in the chamber. The growth enhancement factor (GEF), defined as the ratio of the GR of the total particle population in a charged run to the GR in the corresponding neutral run, was on average about 3 at the size of 1.5 nm and decreased to about 2 at 2 nm for the sulphuric acid–water system (Fig. 3). The magnitude of the enhancement factor corresponds to that of theoretical predictions on the increase in the collision frequency^19^,^20^,^21^. The addition of ammonia to the chamber decreased the GEF slightly, but for experiments with dimethylamine, the enhancement factor was close to unity at both size ranges. This is mainly because particle formation was heavily dominated by neutral mechanisms^25^ and because dimethylamine already stabilizes the clusters effectively, therefore leaving no room for additional stabilization by the ions. On the basis of the results we conclude that the importance of the ion-enhancement is probably low in the atmospheric boundary layer, where stabilizing vapours are usually readily available, but it could be significant in very clean environments, for example, in the free troposphere.

## Discussion

**Figure 4:**
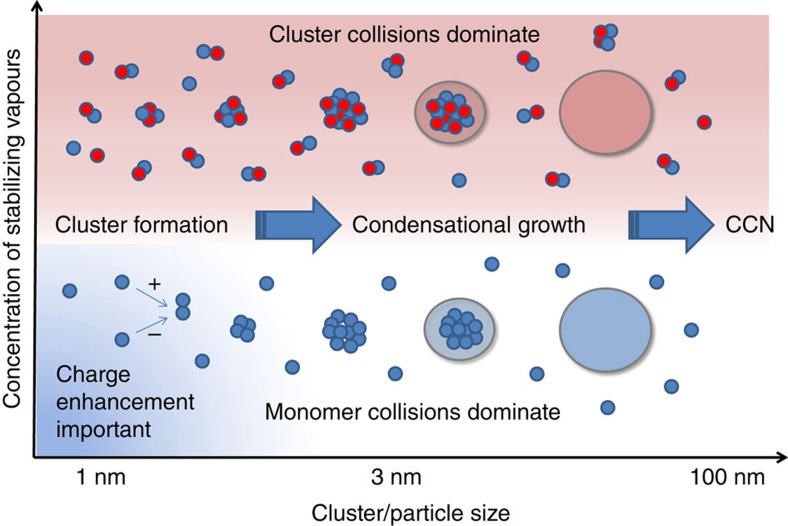
Conceptual summary. Schematics of the factors affecting the growth rate of clusters and particles in a sulphuric acid-driven system. In the presence of vapours which are effective in stabilizing sulphuric acid clusters (for example, ammonia, amines, organic vapours) cluster collisions may assist the growth of clusters to nano-particles and up to CCN, while the enhancement due to electric charge is significant only for the initial steps of cluster formation when the concentrations of stabilizing vapour(s) are low. The absolute concentration required for significant cluster formation depends on the strength of the stabilization.

Figure 4 summarizes our experimental findings and places them into a general framework. The overall growth of nano-particles in the system containing sulphuric acid, water, ammonia or amines and ions appears to be governed by sulphuric acid, but the dominant growth mechanism changes from monomer–cluster collisions, resembling the traditional condensation process, to cluster–cluster collisions when sufficient amounts of strongly basic vapours are present. In the region where base compounds have a limited capability to stabilize the sulphur-containing clusters, the growth is enhanced by electric charges in line with theoretical expectations. The charge effect, however, becomes negligible for sizes above ∼3 nm. In the presence of stabilizing vapours, nano-particle growth is faster due to reduced cluster evaporation rate and is further enhanced by collisions of clusters containing sulphuric acid, which are not detected by conventional chemical ionization mass spectrometers that only measure sulphuric acid vapour.

The significance of this ‘hidden' H_2_SO_4_ in the atmosphere will depend on the cluster distribution under specific conditions, which in turn depends on the cluster formation and loss rates, as well as the properties of the clustering vapours. Clustering is expected to contribute to nano-particle growth over a wide range of atmospheric conditions, especially where the pre-existing particle population is low (Supplementary Fig. 7). This must be taken into consideration when evaluating atmospheric data, especially before drawing any conclusions about the importance of vapours other than sulphuric acid in particle growth. Therefore we recommend that sulphuric acid concentrations and GRs should be measured and modelled using techniques that include the full cluster distribution, especially in areas, where the sulphuric acid concentrations are larger than about 5 × 10^6^ cm^−3^ and amine concentrations are at ppt levels or higher. However, the growth enhancement due to clusters is not limited to an amine system, but is relevant to any system, where sulphuric acid forms clusters with a suitable stabilizing compound. It should be kept in mind that our experiments did not include extremely low-volatility organic compounds known to participate in the new particle formation process in continental boundary layers^3^,^31^,^32^, and that at larger particle sizes organics are still likely to dominate the growth in these environments^12^.

This study has demonstrated that the different nano-particle growth mechanisms in the simultaneous presence of vapour molecules, ions and molecular clusters can be brought to a coherent framework, where, depending on the availability on stabilizing vapours, the nano-particle growth in a sulphuric acid driven system can be assisted either by ions (in the absence or at relatively low concentrations of stabilizing vapours), or by cluster-cluster collisions (in the presence of a strong stabilizing compound, like dimethylamine). These growth enhancements at sub-3 nm sizes can significantly increase the survival of the recently formed clusters to aerosol particles and further to cloud condensation nuclei.

## Methods

### The CLOUD experiment

The CLOUD experiment was designed to study the possible influence of galactic cosmic rays on atmospheric new particle formation. A series of particle formation experiments was performed in a stainless steel 26 m^3^ chamber, which could be exposed to the pion beam from the CERN Proton Synchroton to simulate galactic cosmic rays. Details of the chamber and gas system can be found in Duplissy *et al*.^33^ and references therein. The data presented in this article was collected during the CLOUD3 campaign in October–November 2010, CLOUD4 in June–July 2011, and CLOUD7 in October–December 2012. The particle formation rates (*J*_1.7_) corresponding to the experiment series in Fig. 1 are published by Almeida *et al*.^4^ and they range between about 0.001 and 500 cm^−3^ s^−1^ depending on the gas concentrations used. The numbers of particles participating in the growth process depend on the formation rate, and the maximum concentration reached during the experiment range from about 10 to 10^6^ cm^−3^ (typically 10^2^ to 10^4^ cm^−3^).

### The PSM

The Airmodus A09 Particle Size Magnifier (PSM)^34^ is a dual-stage mixing-type condensation particle counter (CPC), which uses diethylene glycol to activate particles and grow them to about 90 nm in diameter. Further growth to detectable sizes for optical counting was done with a TSI 3010 (CLOUD3 and CLOUD7) or TSI 3772 (CLOUD4) butanol-CPC. The cutoff size (50% activation efficiency) of the PSM was varied between about 1.1 and 2.5 nm by changing the mixing ratio of the sample and saturator flow, which determines the supersaturation inside the instrument. The relation between cutoff size and mixing ratio was determined in laboratory calibrations using mobility standards (tetra-alkyl ammonium halide salts electrosprayed from methanol solutions)^35^ and size-selected silver and ammonium sulphate particles produced with a tube furnace. The size classification was done using a high-resolution Herrmann Differential Mobility Analyzer. The composition of the ions used for calibration was verified after size selection with an APi-TOF mass spectrometer (see below). Details of the calibration set-up and results are discussed elsewhere^36^,^37^. The concentration measured with the PSM was calculated for several different cutoff sizes between about 1 and 3 nm assuming step-wise cutoff functions according to the calibrations, which were used to convert the data into a size distribution^24^. An example of the particle concentrations during an experiment with DMA is given in Supplementary Fig. 1. It should be noted that particle composition can affect their detection efficiency close to the instrument cutoff size. On the basis of calibrations with ions of different compositions and charging states, the error in cutoff diameter is assumed to be in the order of ±0.2 nm for inorganic ions^3^,^24^.

### The APi-TOF

The atmospheric pressure interface time-of-flight mass spectrometer (APi-TOF)^38^ measures the composition and concentrations of ions at mass-to-charge ratios ranging from about 50 Th to 3,300 Th (1 Th=1 Da per *e*, where *e*=electric charge). Only singly charged ions were observed, so mass-to-charge ratio (Th) can be used interchangeably with mass (Da). Sample ions are pulled through a 300-μm orifice and then guided with ion focusing quadrupoles and ion lenses through two consecutive chambers, where the pressure is gradually lowered. In the final TOF chamber (pressure of 10^−6^ mbar) ions are pushed orthogonally to their entrance trajectories to measure the time of flight (flight path about 1 m). The instrument's mass accuracy is better than 10 μTh per Th with mass resolving power 5,000 Th per Th (resolving power is defined as a ratio of mass and full width of the peak at half maximum intensity). During the CLOUD3 campaign, one APi-TOF was used either in negative or positive mode (mostly in negative mode, for detecting negatively charged ions). During the CLOUD4 and CLOUD7 campaigns, two APi-TOFs were deployed; one measuring negatively charged ions continuously, while the other measured positively charged ions.

During the formation of negative and/or positive ions in the CLOUD chamber, the relevant ion spectra measured by the APi-TOFs were dominated by clusters of the shapes (H_2_SO_4_)_*a*_ q^+/−^, (NH_3_)_*b*_ (H_2_SO_4_)_*a*_ q^+/−^, or (C_2_H_7_N)_*b*_ (H_2_SO_4_)_*a*_ q^+/−^, where the ion q^+/−^ would be either HSO_4_^−^ or HSO_5_^−^ for anions, and either NH_4_^+^ or C_2_H_8_N^+^ for cations. Nucleation in the ion channel(s) was either initiated by increasing sulphuric acid concentrations (by increasing ultra violet illumination) or by increasing the ion production rate (by either turning off the chamber's high-voltage clearing field or turning on the beam of ionizing radiation). Any of these actions resulted in an increase of the count rates of all ions. The count rates of ion clusters, however, increased later or more slowly than the count rates of the single molecule ions. The bigger the clusters, the slower their count rates rose. Eventually (within <1 to 20 min), the count rates of all ions reached a ‘steady-state' level.

### The CI-APi-TOF

The chemical ionization atmospheric pressure interface time-of-flight (CI-APi-TOF) mass spectrometer comprises a specially designed inlet for CI at ambient pressure, and the above-described APi-TOF. The instrument is described in detail elsewhere^27^ therefore only a brief discussion of the CI-part of the system is given here.

The CI-system used here is the prototype of Airmodus Ltd chemical ionization source (model: CI-02). The design of the CI-inlet is based largely on the original NCAR-design^27^,^39^,^40^. The largest difference from above-cited systems is the ion production method. Here ions are produced in a sheath flow concentric to the sample flow by <10 keV photons generated by an X-ray tube (Hamamatsu L9490). Minute quantities of nitric acid vapour were fed into the sheath gas resulting in the formation of NO_3_^−^(HNO_3_)_*n*,*n*=0–2_ ions. These ions are mixed with the sample flow entering the ion–molecule interaction chamber at the centre line by means of an electric field. The design is virtually wall-less so losses of ions to the wall occur only in the 12-cm sample inlet tube. The sample flow in the system is 10 l min^−1^ and the concentric sheath flow where ions are produced is 20 l min^−1^. Cryogenic nitrogen was used as sheat gas to minimize possible contamination.

The CI-system was originally designed for measurements of sulphuric acid. Ionization in the CI-system occurs at ambient pressure via a proton transfer between nitrate ions and sulphuric acid:


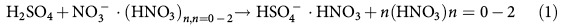


The chemically ionized sample is guided into the APi-TOF through a critical orifice at a flow rate of ∼0.8 l min^−1^. Clusters in equation (1) partly decompose in the vacuum of the APi-TOF. The sulphuric acid concentration (in molecules cm^−3^) measured with the CI-APi-TOF is calculated from the measured ion signals according to:


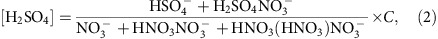


where *C* is the calibration coefficient. The calibration coefficient includes the correction from the losses in the sample line from the CLOUD chamber to the CI-APi-TOF (∼40% transmission). The calibration coefficient of 1.25 × 10^10^ cm^−3^ is obtained using a sulphuric acid generator for calibration^41^. The detection limit (defined here as signal level of three times the s.d. of the background with 15 min integration time) of sulphuric acid monomer has been defined in an earlier study to be 3.2 × 10^4^ cm^−3^ (ref. 27). The error in the sulphuric acid monomer concentration arises mainly from instrument calibrations, and is estimated to be ±45% (ref. 42).

Here the instrument was also used to detect clusters of sulphuric acid and dimethylamine (Supplementary Figs 2 and 4). In case of clusters, the quantification of the exact concentration is more complicated due to lack of established calibration methods. Clusters are not necessarily charged upon every collision with nitrate ions and they are likely to lose an amine molecule on conversion of a sulphuric acid molecule to bisulphate ion (which is essentially a base) in the cluster^41^,^42^,^43^. The clusters can also undergo some evaporation or fragmentation inside the vacuum of the APi-TOF. To protect the clusters, APi-TOF was tuned to cause as little fragmentation as possible, but experimental or theoretical data on the stability would be required to determine the absolute concentrations accurately. For the purposes of this study the exact concentration of neutral clusters is not required as the growth rate can be determined independent of the absolute concentrations. More discussion on the cluster concentration evaluation in the H_2_SO_4_–DMA system can be found in the literature^25^,^26^. Jen *et al*.^26^ also discusses the differences in the detected cluster concentrations using either nitrate or acetate as chemical ionization method in a cluster-CIMS, and shows progress in reconciling the mass spectrometric method with particle number concentration measurements.

### The nano-SMPS

The aerosol size distribution was measured with a scanning mobility particle sizer (nano-SMPS). The differential mobility analyzer of the SMPS was designed to have a higher size resolution and transmission for particle mobility equivalent diameters between 5 and 90 nm. The particle concentration behind the differential mobility analyzer was measured with a modified TSI 3772 CPC.

### The NAIS

The ion concentration and size distribution in the CLOUD chamber was measured with a neutral cluster and air ion spectrometer (NAIS)^44^. The NAIS is able to measure the ion number size distributions in the mobility equivalent diameter range of 0.8 to 40 nm and the total particle number size distributions from about 2 to 40 nm in mobility diameter. The instrument used in CLOUD was a second generation version of the instruments (so called Airborne NAIS) with improvements especially on the flow control^45^.

### Measurements of the gas phase compounds

The sulphuric acid concentration was measured with a CIMS^40^, channel 97 Th. In CLOUD7, also the CI-API-TOF could be used to determine the sulphuric acid (monomer) concentration. Ammonia was measured in CLOUD3 by long path absorption spectrophotometry^22^ and a proton transfer reaction mass spectrometer (PTR-MS)^46^. In CLOUD4 and CLOUD7 ammonia concentrations were measured with a PTR-MS and an ion chromatograph^23^, which additionally measured the dimethylamine concentration. Proton transfer reaction time of flight (PTR-ToF)^47^ mass spectrometer was used for measurements of organic vapour concentrations in the chamber^48^.

### The appearance time method for determining GRs

There is no established method available to study the growth rate of total (or neutral) particle population in the size range below 3 nm. At the very beginning of the particle formation process, the particle population does not yet form a distinct ‘mode', so the mode fitting or maximum concentration methods^49^,^50^ frequently used for size distributions at larger sizes were not directly applicable. Therefore, we developed the appearance time method^24^, which can be used for obtaining information about the growth rate even at the beginning of the growth process. In this method, we search for the time when certain sized clusters are formed and the growth rate can be determined based on the time lag between the formation of successive clusters.

The appearance time method resembles the time lag method of Riccobono *et al*.^51^ where they estimated GRs based on the time difference of particle detection in different CPCs at different cutoff sizes. However, by adjusting the cutoff size of one instrument, instead of comparing different instruments, we could avoid the systematic error in the time lag method arising from different shapes of the instrument cutoff curves^52^. Also, the appearance time method can be applied to particle data in narrow size bins, or even single clusters. This method was used here for the data measured with the (CI)-APi-TOF and the PSM. The appearance times from the PSM and the CI-APi-TOF for a series of experiments with H_2_SO_4_ and DMA is presented in Supplementary Fig. 2.

Modelling studies^24^,^52^ on how well the appearance time growth rate represents the actual growth rate of the particles indicate that the appearance time method can give larger values especially at the beginning of the growth process and for very small clusters, but the values approach the ‘reference growth rate' (the input GR in the aerosol dynamic simulation^24^) before about 2 nm. The appearance time GR was also shown to approach the GR calculated from the molecular fluxes with increasing cluster size, but the agreement between these two quantities depended strongly both on the model system and the external conditions^52^. Situations where cluster–cluster collisions contributed significantly to the growth were not considered in the comparison. Both the cluster properties (charge distribution, evaporation rate and so on) and their dynamics (formation rate, loss rate) affect how robustly the growth rate can be determined, which is also true for most other methods to determine GRs from measurement data^21^.

### GRs from the PSM

The growth rate was determined from the appearance times of the particle population with different cutoff sizes of the PSM. The appearance times were plotted against the cutoff size, and the GR was determined from a linear fit between the times and sizes as in Supplementary Fig. 2. The fitting was done separately for the size range 1–2 nm (GR_1.5_) and 1.5–2.5 nm (GR_2_).

The appearance time was defined either as the time when the first particles appeared to a certain cutoff size (∼5% increase in the total concentration), or as the time when the concentration in a size bin reached 50% of its maximum value. Both of these alternatives gave approximately same results for the GR. The robustness of the method under a range of conditions has been tested in a sensitivity study^24^, which indicated that the resulting GRs are quite insensitive to the way of selecting the appearance time or small errors in determining the cutoff curves.

Since the time resolution of the PSM in scanning mode is 2 min, this method does not give a reasonable value for runs with very high growth rate (>30 nm h^−1^), as the particles appear almost simultaneously in all size ranges, and these cases were excluded from the analysis. Using this method, a GR value could be calculated from 158 individual experiments for the CLOUD3 campaign, 109 for the CLOUD4 campaign and 90 for CLOUD7 campaign.

### GRs from the APi-TOF

The data from APi-TOFs were processed with the latest versions of tofTools^38^, a set of programs based on MATLAB. To retrieve ion growth rates, each experiment was analysed separately: the average spectrum over the steady-state periods was used to calibrate the mass axis (that is, the conversion from time-of-flight to mass), and for creating a peak list, including identified as well as unidentified compounds. The data of interest was then averaged over 0.5 min time steps, and the mass axis calibration applied to each 0.5 min spectrum. Counts therein were attributed to appropriate peaks from the peak list, yielding a time series for each peak. For obtaining growth rates of negatively charged ions, counts were used from (H_2_SO_4_)_*a*_ HSO_4_^−^ (*a*⩾0), (NH_3_)_*b*_ (H_2_SO_4_)_*a*_ HSO_4_^−^ (*b*⩾1, *a*⩾3), and (C_2_H_7_N)_*b*_ (H_2_SO_4_)_*a*_ HSO_4_^−^ (*b*⩾1, *a*⩾2). For obtaining growth rates of positively charged ions, counts were used from (NH_3_)_*b*_ (H_2_SO_4_)_*a*_ NH_4_^+^, and (C_2_H_7_N)_*b*_ (H_2_SO_4_)_*a*_ C_2_H_8_N^+^. Following Ehn *et al*.^53^, a mobility diameter was calculated for each of these compounds, depending only on mass and density of the compounds. Bulk densities of ammonium bisulphate (1,780 kg m^−3^) or sulphuric acid (1,840 kg m^−3^) or linear interpolations in-between were used according to ratios of NH_3_ and H_2_SO_4_ molecules in the compounds. The same was done for mixed C_2_H_7_N/H_2_SO_4_ clusters, while the bulk density of dimethylaminium bisulphate, however, is not known. Densities between 1,100 and 1,900 kg m^−3^ were tested. The differences between the resulting mobility diameters were small, and a density of 1,500 kg m^−3^ was assumed thereafter. All clusters with the same number of H_2_SO_4_ molecules, but varying base content were combined into one data point for the line fitting to improve the signal-to-noise ratio of count rates. Each time series was normalized to the steady-state count rates before the start of the experiment (=0%) and to the steady-state count rates during new particle formation (=100%), and smoothed (moving average over 2 min). The points in time (at 0.5 s precision) when each of these time series passed through 50% were plotted versus the corresponding mobility diameters. The inverse of the slope of an applied linear fit equalled the growth rate. A similar method was used for the CI-APi-TOF.

### Comparison to other methods for determining GRs

The GRs of charged particle (negative and positive ions) were determined for three size ranges (below 3, 3–7 nm and larger than 7 nm) from size distributions measured by the NAIS using the maximum-concentration method^49^,^50^. Following this method, we determined the time when the maximum concentration in each size fraction of the instrument was reached. To minimize the influence of noise in the data, the maximum time was determined from fitting a Gaussian function at the approximate location of the concentration maximum, and using the peak of this fitted function as the maximum time. The particle growth rate was then obtained as the slope of a linear least-squares fit to the data points of the geometric mean diameter of the size fraction versus time of maximum concentration.

The growth rates at sizes larger than 3 nm were determined from the measured particle size distribution either by the mode fitting method or by following the edge of the distribution. These methods were used for particle size distributions measured by the nano-SMPS or by the NAIS in total particle mode, and for the combination of a nano-radial-differential mobility analyzer (nRDMA) and a PSM. Also the size information given by the laminar diffusion tube, which combines an ultrafine CPC with the particle size-dependency of the diffusion losses in the sampling line, was used for estimating the growth rate.

Supplementary Fig. 3 compares the GRs from the CLOUD4 campaign determined from the different instruments both with and without dimethylamine in the chamber. In general the agreement is good between the different instruments, even though they have slightly different size ranges, and the GR data were analysed with different methods. The apparent increase in GR due to dimethylamine can be seen with all the methods, which rules out any artefact in the GR measurement due to particle composition.

### The GEF

The GEF was defined as the ratio between the total particle population growth rate in a charged run with the pion beam (or galactic cosmic rays run if GR for the corresponding charged run could not be determined) divided by the total particle population growth rate in a neutral run, as described in equation (3). In the charged run the conditions were otherwise exactly similar to the neutral run, but there were ions present in the chamber.


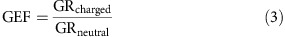


The determined GEFs should be considered maximum estimates. As the sulphuric acid concentration does not immediately rise as a step function when ultra violet light is turned on, it might be still rising in the beginning of the neutral run, whereas the charged runs were usually started when the sulphuric acid concentration had already stabilized. This would especially affect the lowest size when GR is high, and thus give a slightly too high GEF. To minimize this artefact, we calculated the mean of the sulphuric acid concentration for each experiment during the exact period from which the GR was determined, and if the concentration differed by more than 10% in the charged and neutral run, the pair was excluded from the GEF analysis.

It should be noted that the GEF does not describe the difference between the growth rate of an individual neutral particle and an individual ion, but rather the effect of the ions onto the apparent growth rate of the total population, which is affected by the charging state of the aerosol population and the ion and aerosol dynamics^21^.

### The simulated molecular clusters

The effect of neutral clusters on the nano-particle growth rates was modelled with atmospheric cluster dynamics code^28^. Instead of studying the full four-component acid–ammonia–DMA–water system, the simulations were performed on a simplified quasi-unary model system with single-component clusters containing up to 70 molecules. The model substance consisted of spherical molecules with the properties of the sulphuric acid–dimethylamine dimer: a molecular mass of 143.16 a.m.u. and an assumed liquid density of 1,500 kg m^−3^. In all the simulations, the temperature was set to 278 K. In cluster evaporation rate calculations, a surface tension of 0.05 N m^−1^ was used, and the saturation vapour pressure was lowered from that of sulphuric acid to values between 5 × 10^−9^ and 10^−7^ Pa to qualitatively mimic the stabilization of clusters by base molecules.

The time evolution of the cluster concentrations was obtained by numerically solving the time derivatives of the concentrations *C*_*i*_ of all the modelled cluster sizes *i*=1–70


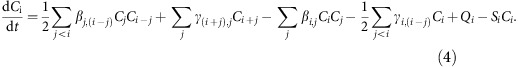


Here *β*_*i*,*j*_ is the collision coefficient for the collision of clusters *i* and *j*, *γ*_(*i*+*j*)→*i*,*j*_ is the evaporation rate of cluster *i*+*j* breaking into clusters *i* and *j*, and *S*_*i*_ is the loss rate of cluster *i* onto the walls of the chamber. *Q*_*i*_ is a production rate relevant only for the monomer *i*=1. The terms on the right-hand-side include all possible collisions and evaporations that form or destroy cluster *i*: those occurring between a cluster and a vapour monomer, and those involving two clusters, or two monomers. The evaporations, however, were not included in all the simulation sets (see section The collision and evaporation rates in the simulations below). Collisions products larger than the 70-mer were lost from the simulated system and assumed not to evaporate back to smaller sizes.

### Determining the growth rates from the simulations

The growth rates in all model runs were determined by using a similar method as with the measured data: the appearance times (50% of total concentration) of different sized clusters were determined and a linear fit was applied to times and cluster sizes to obtain the growth rate at c. 2 nm mobility diameter. The fit was applied to a size range of five adjacent clusters around the size of 2 nm. The obtained growth rates are presented as a function of the average monomer concentration over the appearance time period. Supplementary Fig. 5 illustrates the method used for the simulation data using the same average monomer concentration during the growth time as in the experiments presented in Supplementary Fig. 2 and assuming zero evaporation rate of the clusters. Note that in this study we considered the enhancement in GR compared with the same sulphuric acid monomer concentration and not the total sulphuric acid concentration, which includes also the acid molecules bound to clusters. The relation between modelled total sulphuric acid and the measured sulphuric acid (monomer or total) concentration in the dimethylamine experiments from CLOUD is studied in more detail by Rondo *et al*.^30^.

### The collision and evaporation rates in the simulations

The collision rates *β* were calculated as hard-sphere collisions from kinetic gas theory, and the evaporation rates *γ* were determined via detailed balance from the Gibbs free energies of the clusters calculated according to the liquid drop model. Detailed formulae for the rate coefficients and the free energies can be found in the literature^28^,^52^, respectively. As one of our aims was to study how cluster stabilization affects the appearance time growth rates, we performed several simulation sets using different values for the evaporation rates. In Fig. 1, we show a set of simulations with all evaporation rates set to zero. Supplementary Fig. 6 shows the effect of evaporation rates of different orders onto the appearance time growth rates.

The assumption of spherical droplets with the bulk liquid density in the collision coefficient calculation gives rise to some uncertainty in the collision cross-sections. Furthermore, dipole–dipole interactions (or to a lesser extent Debye forces and London dispersion forces) can increase the collision rates compared with the hard-sphere approximation. On the other hand, all collisions might not stick, and a sticking factor lower than unity has the same effect as lowering the collision rate. This uncertainty in the (effective) collision rates was studied by running two additional sets of simulations for Fig. 1: one with all collision coefficients multiplied by three and another with all rates divided by two. This uncertainty range gives the grey shaded area in Fig. 1. The kinetic model used by Kürten *et al*.^25^ to simulate cluster formation in a DMA–sulphuric acid system uses an enhancement factor of about 2.3 for the collision rates and gave identical GRs, when using the same assumptions and the same input parameters.

### The scavenging of clusters in the simulations

The wall-loss rate constants *S* were calculated according to the formula presented by Almeida *et al*.^4^. The magnitude and size dependence of the wall losses affect the time evolution of the cluster concentrations and thereby the appearance time growth rate, so the uncertainty in the wall-loss parameterization is propagated to an uncertainty in the modelled GRs.

To evaluate in which kind of atmospheric conditions we can expect the effect of clustering on the particle growth rates to be significant, we performed additional sets of simulations varying the magnitude of the loss rates. In these simulations the size-dependency of the loss rate was assumed to be of the form


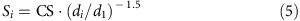


where CS=*S*_1_ is the condensation sink of the vapour monomer *i*=1, *d*_*i*_ is the geometric diameter of cluster *i* and *d*_1_ is the monomer diameter. This corresponds to a typical size-dependency of the sink caused by a population of larger aerosol particles in the atmosphere. The growth rates were calculated using different values of vapour condensation sink CS from 10^−4^ s^−1^ representing a very clean environment to 10^−2^ s^−1^ representing a quite polluted environment. For each value of condensation sink, simulations were performed for sulphuric acid production rates between 10^3^ and 10^7^ cm^−3^ s^−1^. The results are presented in Supplementary Fig. 7 as a function of the resulting average acid concentrations.

As expected, a higher condensation sink lowers the GRs in the case with significant clustering. On the other hand, when evaporation is considered, the GRs increase with increasing CS (although the number of clusters participating in the growth decreases with increasing CS). The increase in the GR is related to the cluster distribution reaching steady-state faster. A similar effect was observed by Olenius *et al*.^52^ both for the flux GR and for the appearance time GR studied here. For the flux GR, this phenomenon can be understood by considering the shape of the cluster distribution. The sink lowers the concentration of all clusters, but the effect is stronger for larger clusters, and therefore the backward (evaporation) flux decreases more than the forward (collision) flux, increasing the flux GR. As a result, an increasing CS brings the GRs for an evaporating system closer to the case with no evaporation, although the GRs are still higher when there is no evaporation. This indicates that the significance of clusters to GR gets smaller, but not negligible with increasing sink. Even at a high condensation sink (CS 10^−2^ s^−1^), typical for highly polluted environments, there is a region, where clusters clearly affect the GR.

## Additional information

**How to cite this article:** Lehtipalo, K. *et al*. The effect of acid–base clustering and ions on the growth of atmospheric nano-particles. *Nat. Commun.* 7:11594 doi: 10.1038/ncomms11594 (2016).

## Supplementary Material

Supplementary Information Supplementary Figures 1-7 and Supplementary References.

## References

[b1] Merikanto J., Spracklen D. V., Mann G. W., Pickering S. J. & Carslaw K. S. Impact of nucleation on global CCN. Atmos. Chem. Phys. 9, 8601–8616 (2009).

[b2] Kirkby J. . Role of sulphuric acid, ammonia and galactic cosmic rays in atmospheric aerosol nucleation. Nature 476, 429–433 (2011). 21866156 10.1038/nature10343

[b3] Kulmala M. . Direct observations of atmospheric aerosol nucleation. Science 22, 911–912 (2013). 10.1126/science.1227385 23430652

[b4] Almeida J. . Molecular understanding of sulphuric acid-amine particle nucleation in the atmosphere. Nature 502, 359–3663 (2013). 24097350 10.1038/nature12663 PMC7449521

[b5] Schobesberger S. . Molecular understanding of atmospheric particle formation from sulphuric acid and large oxidized organic molecules. Proc. Natl Acad. Sci. 110, 17223–17228 (2013). 24101502 10.1073/pnas.1306973110 PMC3808659

[b6] Riipinen I. . Connections between atmospheric sulphuric acid and new particle formation during QUEST III–IV campaigns in Heidelberg and Hyytiälä. Atmos. Chem. Phys. 7, 1899–1914 (2007).

[b7] Lehtipalo K. . Nanoparticles in boreal forest and coastal environment: a comparison of observations and implications of the nucleation mechanism. Atmos. Chem. Phys. 10, 7009–7016 (2010).

[b8] Kuang C. . Size and time-resolved growth rate measurements of 1 to 5 nm freshly formed atmospheric nuclei. Atmos. Chem. Phys. 12, 3573–3589 (2012).

[b9] Sipilä M. . The role of sulphuric acid in atmospheric nucleation. Science 327, 1243–1246 (2010). 20203046 10.1126/science.1180315

[b10] Nieminen T., Lehtinen K. E. J. & Kulmala M. Sub-10 nm particle growth by vapour condensation – effects of vapour molecule size and particle thermal speed. Atmos. Chem. Phys. 10, 9773–9779 (2010).

[b11] Metzger A. . Evidence for the role of organics in aerosol particle formation under atmospheric conditions. Proc. Natl Acad. Sci. 107, 6646–6651 (2010). 20133603 10.1073/pnas.0911330107 PMC2872387

[b12] Riipinen I. . The contribution of organics to atmospheric nanoparticle growth. Nature Geosci. 5, 453–458 (2012).

[b13] Smith J. N. . Chemical composition of atmospheric nanoparticles formed from nucleation in Tecamac, Mexico: Evidence for an important role for organic species in nanoparticle growth. Geophys. Res. Lett. 35, L04808 (2008).

[b14] Bzdek B. . Quantitative and time-resolved nanoparticle composition measurements during new particle formation. Faraday Discuss. 165, 25–43 (2013). 24600995 10.1039/c3fd00039g

[b15] Smith J. N. . Observations of aminium salts in atmospheric nanoparticles and possible climatic implications. Proc. Natl Acad. Sci. 15, 6634–6639 (2010). 20080626 10.1073/pnas.0912127107 PMC2872393

[b16] Erupe M. E., Viggiano A. A. & Lee S.-H. The effect of trimethylamine on atmospheric nucleation involving H_2_SO_4_. Atmos. Chem. Phys. 11, 4767–4775 (2011).

[b17] Chen M. . Acid–base chemical reaction model for nucleation rates in the polluted atmospheric boundary layer. Proc. Natl Acad. Sci. 109, 18713–18718 (2012). 23091030 10.1073/pnas.1210285109 PMC3503223

[b18] Yu H., McGraw R. & Lee S.-H. Effects of amines on formation of sub-3 nm particles and their subsequent growth. Geophys. Res. Lett. 39, L02807 (2012).

[b19] Laakso L., Kulmala M. & Lehtinen K. E. J. Effect of condensation rate enhancement factor on 3-nm (diameter) particle formation in binary ion-induced and homogeneous nucleation. J. Geophys. Res. 108, 4574 (2003).

[b20] Nadykto A. B. & Yu F. Uptake of neutral polar vapour molecules by charged clusters/particles: Enhancement due to dipole-charge interaction. J. Geophys. Res. 108, 4717 (2003).

[b21] Leppä J., Anttila T., Kerminen V.-M., Kulmala M. & Lehtinen K. E. J. Atmospheric new particle formation: real and apparent growth of neutral and charged particles. Atmos. Chem. Phys. 11, 4939–4955 (2011).

[b22] Bianchi F., Dommen J., Mathot S. & Baltensperger U. On-line determination of ammonia at low pptv mixing ratios in the CLOUD chamber. Atmos. Meas. Tech. 5, 1719–1725 (2012).

[b23] Praplan A. P., Bianchi F., Dommen J. & Baltensperger U. Dimethylamine and ammonia measurements with ion chromatography during the CLOUD4 campaign. Atmos. Meas. Tech. 5, 2161–2167 (2012).

[b24] Lehtipalo K. . Methods for determining particle size distribution and growth rates between 1 – 3 nm using the particle size magnifier. Boreal Environ. Res. 19, 215–236 (2014).

[b25] Kürten A. . Neutral molecular cluster formation of sulfuric acid–dimethylamine observed in real time under atmospheric conditions. Proc. Natl. Acad. Sci. USA 111, 15019–15024 (2014). 25288761 10.1073/pnas.1404853111 PMC4210346

[b26] Jen C. N., Hanson D. R. & McMurry P. H. Toward reconciling measurements of atmospherically relevant clusters by chemical ionization mass spectrometry and mobility classification/vapor concentration. Aerosol Sci.Technol. 49, i–iii (2015).

[b27] Jokinen T. . Atmospheric sulphuric acid and neutral cluster measurements using CI-APi-TOF. Atmos. Chem. Phys. 12, 4117–4125 (2012).

[b28] McGrath M. J. . Atmospheric cluster dynamics code: a flexible method for solution of the birth-death equations. Atmos. Chem. Phys. 12, 2345–2355 (2012).

[b29] McMurry P. H. Photochemical aerosol formation from SO2: a theoretical analysis of smog chamber data. J. Colloid Interface Sci. 78, 513–527 (1980).

[b30] Rondo L. . Effect of dimethylamine on the gas phase sulfuric acid concentration measured by Chemical Ionization Mass Spectrometry. J. Geophys. Res. Atmos. 121, 3036–3049 (2016). 10.1002/2015JD023868 PMC4996328 27610289

[b31] Ehn M. . A large source of low-volatility secondary organic aerosol. Nature 506, 476–479 (2014). 24572423 10.1038/nature13032

[b32] Riccobono F. . Oxidation products of biogenic emissions contribute to nucleation of atmospheric particles. Science 344, 717–721 (2014). 24833386 10.1126/science.1243527

[b33] Duplissy J. . Effect of ions on sulfuric acid-water binary particle formation: 2. Experimental data and comparison with QC-normalized classical nucleation theory. J. Geophys. Res. Atmos. 121, 1752–1775 (2016).

[b34] Vanhanen J. . Particle size magnifier for nano-CN detection. Aerosol Sci.Technol. 4, 533–542 (2011).

[b35] Ude S. & Fernández de la Mora J. Molecular monodisperse mobility and mass standards from electrosprays of tetra-alkyl ammonium halides. J. Aerosol Sci. 36, 1224–1237 (2005).

[b36] Kangasluoma J. . Remarks on ion generation for CPC detection efficiency studies in sub 3 nm size range. Aerosol Sci. Technol. 47, 556–563 (2013).

[b37] Wimmer D. . Performance of diethylene glycol-based particle counters in the sub-3 nm size range. Atmos. Meas. Tech. 6, 1793–1804 (2013).

[b38] Junninen H. . A high-resolution mass spectrometer to measure atmospheric ion composition. Atmos. Meas. Tech. 3, 1039–1053 (2010).

[b39] Eisele F. L. & Tanner D. J. Measurement of the gas phase concentration of H_2_SO_4_ and methane sulfonic acid and estimates of H_2_SO_4_ production and loss in the atmosphere. J. Geophys. Res. 98, 9001–9010 (1993).

[b40] Kürten A., Rondo L., Ehrhart S. & Curtius J. Performance of a corona ion source for measurement of sulphuric acid by chemical ionization mass spectrometry. Atmos. Meas. Tech. 4, 437–443 (2011).

[b41] Kürten A., Rondo L., Ehrhart S. & Curtius J. Calibration of a chemical ionization mass spectrometer for the measurement of gaseous sulfuric acid. J. Phys. Chem. A 116, 6375–6386 (2012). 22364556 10.1021/jp212123n

[b42] Berndt T. . Gas-phase ozonolysis of selected olefins: the yield of stabilized criegee intermediate and the reactivity toward SO_2_. J. Phys. Chem. Lett. 3, 2892–2896 (2012).

[b43] Kurtén T. . The effect of H2SO4 – amine clustering on chemical ionization mass spectrometry (CIMS) measurements of gas-phase sulphuric acid. Atmos. Chem. Phys. 11, 3007–3019 (2011).

[b44] Manninen H. E. . Long-term field measurements of charged and neutral clusters using Neutral cluster and Air Ion Spectrometer (NAIS). Boreal Environ. Res. 14, 591–605 (2009).

[b45] Mirme S. . Atmospheric sub-3nm particles at high altitudes. Atmos. Chem. Phys. 10, 437–451 (2010).

[b46] Norman M., Hansel A. & Wisthaler A. O^+^_2_ as reagent ion in the PTR-MS instrument: Detection of gas-phase ammonia. Int. J. Mass Spectrom. 265, 382–387 (2007).

[b47] Graus M., Mueller M. & Hansel A. High resolution PTR-TOF: quantification and formula confirmation of VOC in real time. J. Am. Soc. Mass Spectrom. 21, 1037–1044 (2010). 20335047 10.1016/j.jasms.2010.02.006

[b48] Schnitzhofer R. . Characterisation of organic contaminants in the CLOUD chamber at CERN. Atmos. Meas. Tech. 7, 2159–2168 (2014).

[b49] Hirsikko A. . Annual and size dependent variation of growth rates and ion concentration in boreal forest. Boreal Environ. Res. 10, 357–369 (2005).

[b50] Yli-Juuti T. . Growth rates of nucleation mode particles in Hyytiälä during 2003–2009: variation with particle size, season, data analysis method and ambient conditions. Atmos. Chem. Phys. 11, 12865–12886 (2011).

[b51] Riccobono F. . Contribution of sulphuric acid and oxidized organic compounds to particle formation and growth. Atmos. Chem. Phys. 12, 9427–9439 (2012).

[b52] Olenius T. . Growth rates of atmospheric molecular clusters based on appearance times and collision–evaporation fluxes: growth by monomers. J. Aerosol Sci. 78, 55–70 (2014).

[b53] Ehn M. . Comparing mobility and mass measurements of atmospheric small ions. Aerosol Sci. Technol. 45, 522–532 (2011).

