# Supplementary material for: The effect of acid–base clustering and ions on the growth of atmospheric nano-particles
Source: Nat Commun. 2016 May 20;7:11594. doi: 10.1038/ncomms11594 (PMC4876472; doi:10.1038/ncomms11594)
Supplement: Supplementary Information — Supplementary Figures 1-7 and Supplementary References. [file ncomms11594-s1.pdf]

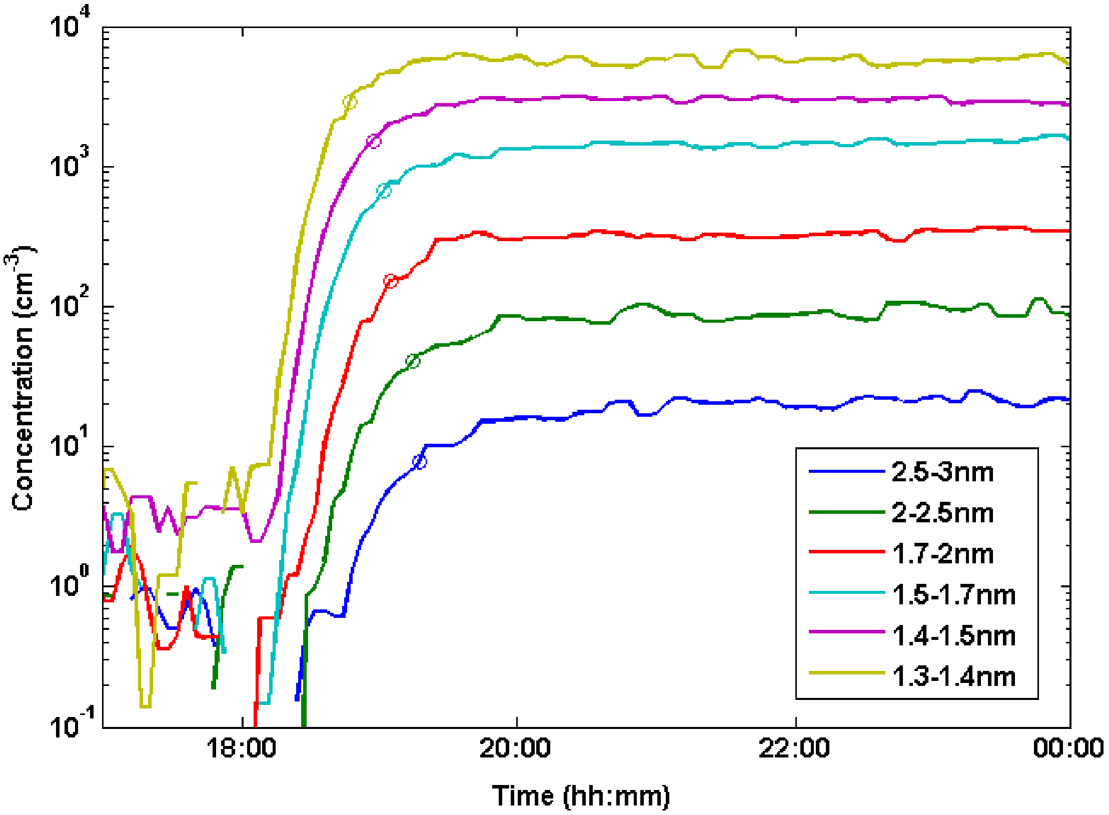

Supplementary Figure 1. Example of a new particle formation experiment in the CLOUD chamber. The different coloured lines are the particle concentration in the size bins calculated based on measurements with the Particle Size Magnifier (PSM) in an experiment with $[H_2SO_4]=2.5\cdot10^6$ cm^{-3} and [DMA]=26 pptv. The small circles denote the time steps when the concentration in each size bin has risen to 50% of its maximum value, which were used for determining the GRs.

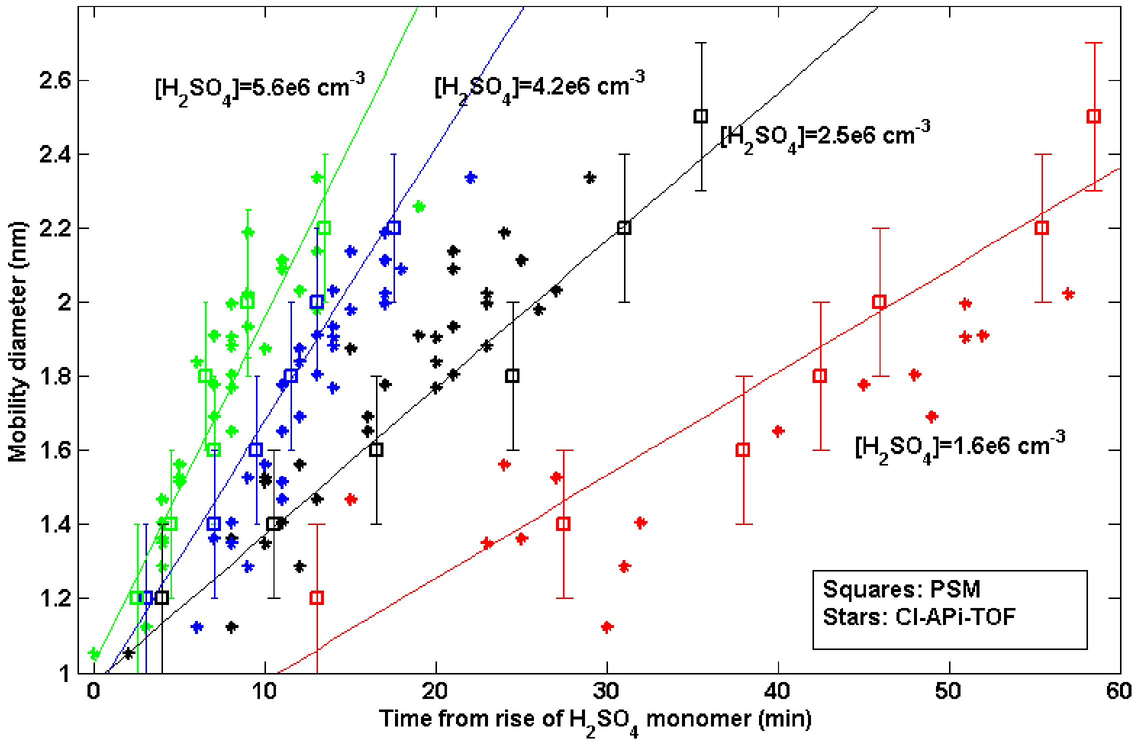

Supplementary Figure 2. Measured appearance times of different sized clusters. The growth of clusters in four neutral experiments with [DMA]=25 pptv and varying H_2SO_4 concentration (given in the figure). Squares are the appearance times of the particle population at the given cut-off diameter of the Particle Size Magnifier, while stars denote the appearance times of different sulphuric acid-amine clusters detected with the CI-APi-TOF mass spectrometer. The appearance time was defined as the time step when the signal reached 50% of maximum. The mass of the clusters was here converted to mobility diameter assuming constant density of 1.6 g cm^{-3}. The error bars denote the +/- 0.2 nm error estimation in the PSM cut-off sizes. The lines are linear fits to the PSM data, yielding the growth rate of particles in each experiment. At higher sulphuric acid concentration the growth was faster, so the slope is higher. Note that for the actual data analysis, the fitting was done separately for the size range 1-2 nm ($GR_{1.5}$) and 1.5-2.5 nm (GR_2).

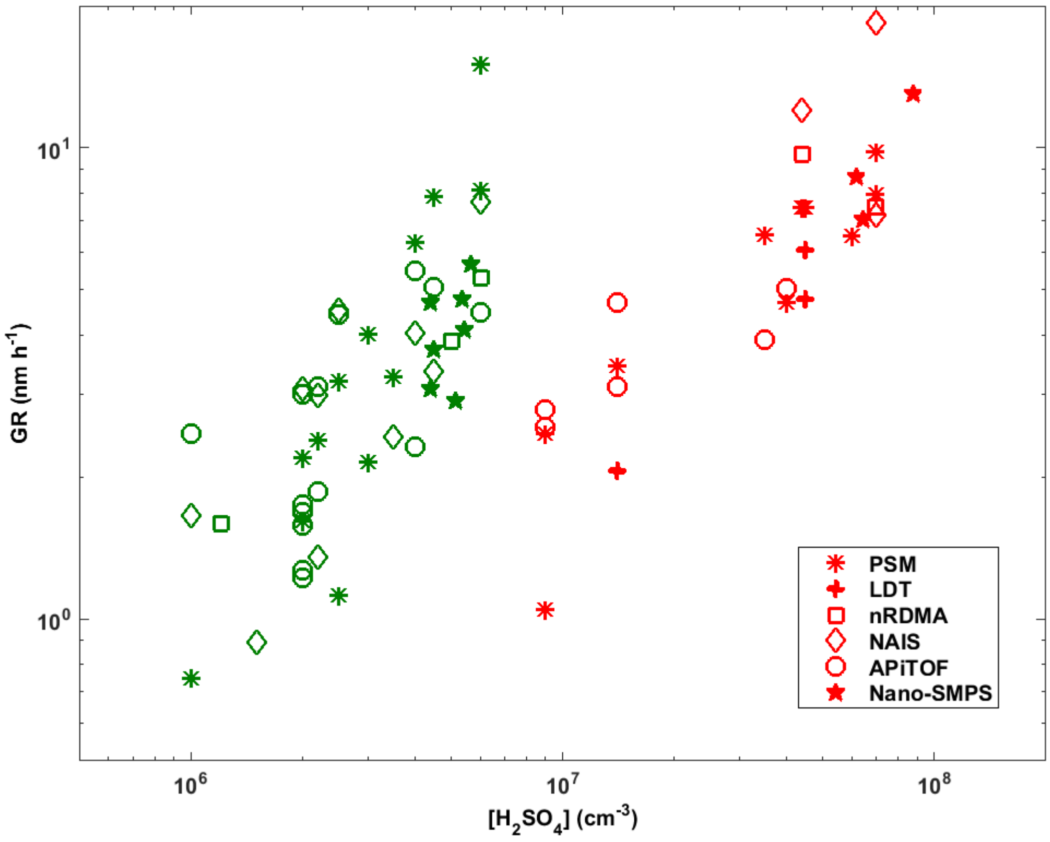

Supplementary Figure 3. Growth rate measurement using different instruments. The growth rates determined from different instruments during the CLOUD4 campaign as a function of sulphuric acid monomer concentration either before the addition of DMA (red symbols) or after addition of >5 pptv DMA (green symbols). The different instruments used for measuring the growth rate are denoted with different symbols (acronyms explained in the text).

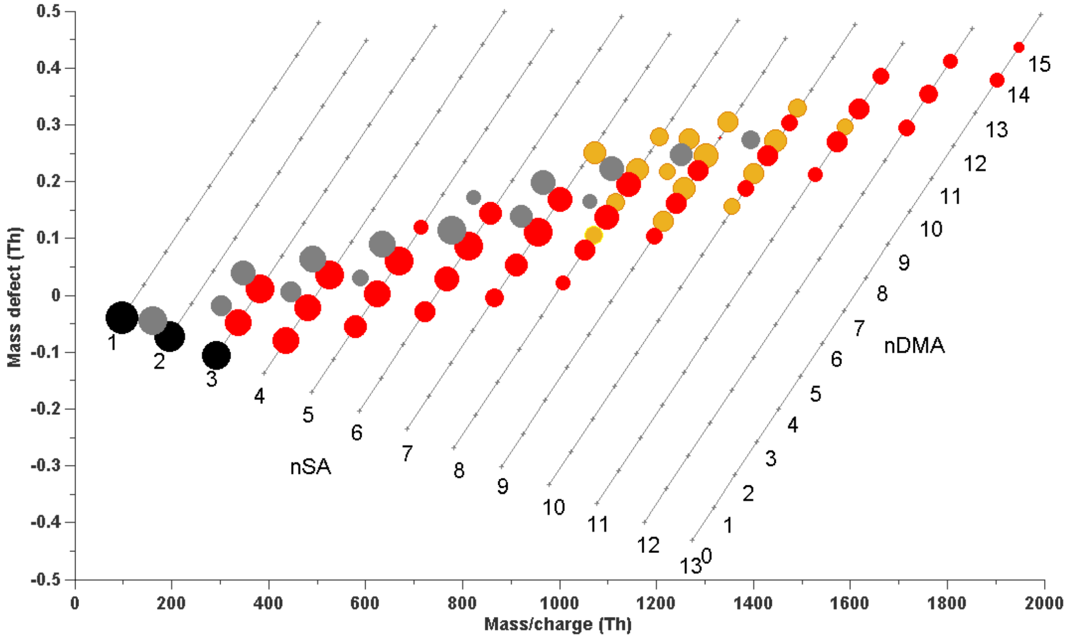

Suppelmentary Figure 4. Neutral clusters observed in DMA experiments. The mass defect plot of clusters measured with the CI-APiTOF during a neutral experiment with 33 pptv DMA (adapted from Kürten *et al.*[1]). The mass defect plot reveals the different atom content in the measured clusters, as oxygen and sulphur have negative mass defects while hydrogen has a strong and nitrogen a weak positive defect. Mass defect is the total deviation of the molecule/cluster mass from the integer mass defined as the sum of neutrons and protons in the atom nuclei of the molecules. The carbon mass defect is zero by definition. Numbers in the figure depict the number of sulphuric acid (nSA) and dimethyl amine (nDMA) molecules in the cluster. Diameters of the markers represent the observed signal intensity in a logarithmic scale. Red markers are used for clusters with sulphuric acid and DMA, grey for sulphuric acid and DMA clustered with NO_3^- or HNO_3, orange for clusters with sulphuric acid, DMA and ammonia and black for molecules and clusters consisting only of sulphuric acid. At least one molecule of dimethyl amine is most likely lost upon ionization of the smallest clusters. For example, the

cluster observed at mass 194.927 corresponding to the sulphuric acid dimer, most likely contained dimethyl amine prior to charging, although the direct information is lost. In this experiment the growth of the sulphuric acid – DMA clusters up to a size of 13 SA and 15 DMA molecules (observed at mass 1948.4 Da) was clearly detectable.

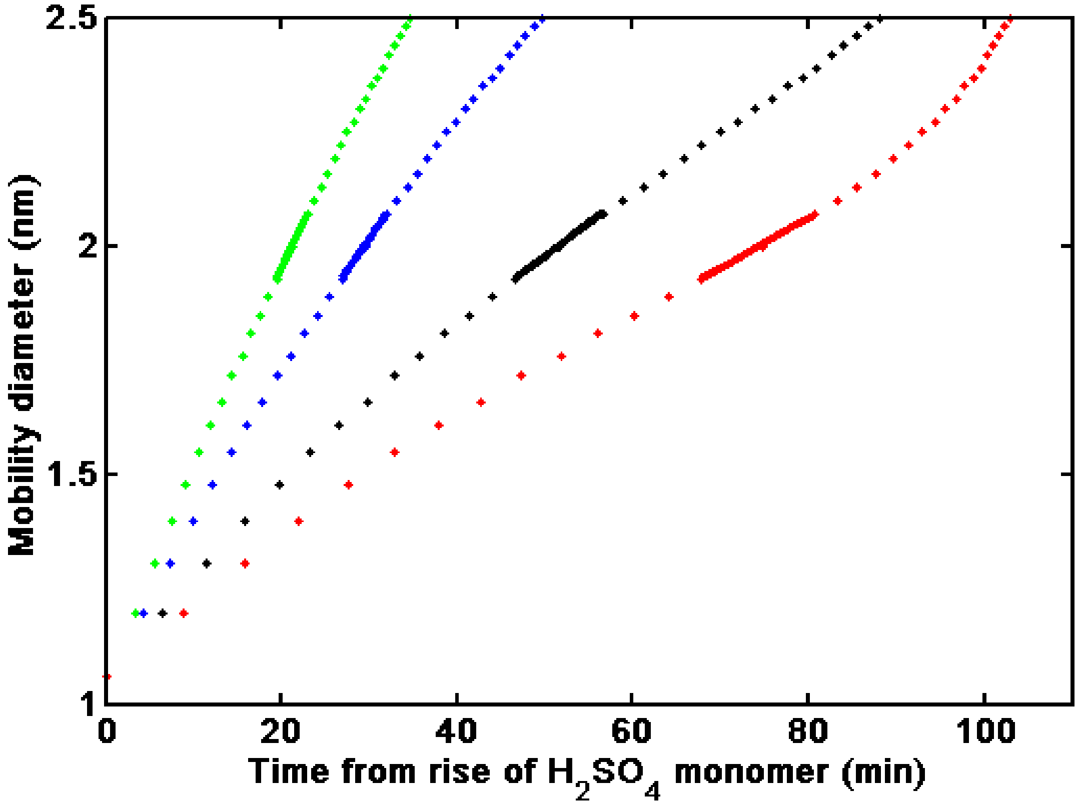

Supplementary Figure 5. Modelled appearance times. An example of how the appearance time growth rates were determined from the cluster population simulations (ACDC). Each dot represents the 50% rise time of a cluster, and a linear fit (solid lines) was applied to 5 successive clusters around the size of 2 nm to determine the GR. The sulphuric acid concentrations used in the simulations correspond to the ones in Supplementary Fig. 2 (same color coding). Note that no enhancement factor was used for the collision rates in these simulations.

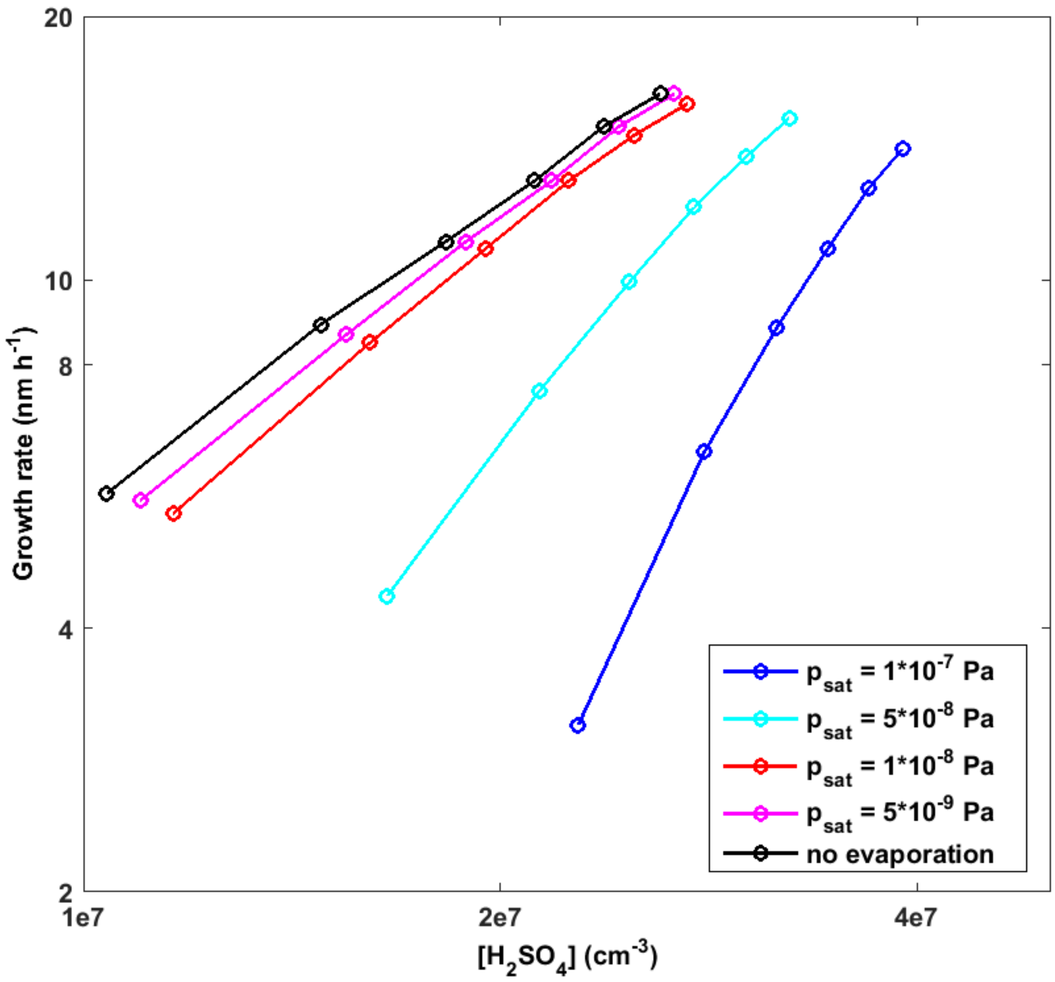

Supplementary Figure 6. Effect of saturation vapor pressure. The simulated appearance time growth rates as a function of sulphuric acid monomer concentration with different saturation vapour pressures P_{sat} of the model substance. The cluster evaporation rates are directly proportional to the saturation vapour pressure.

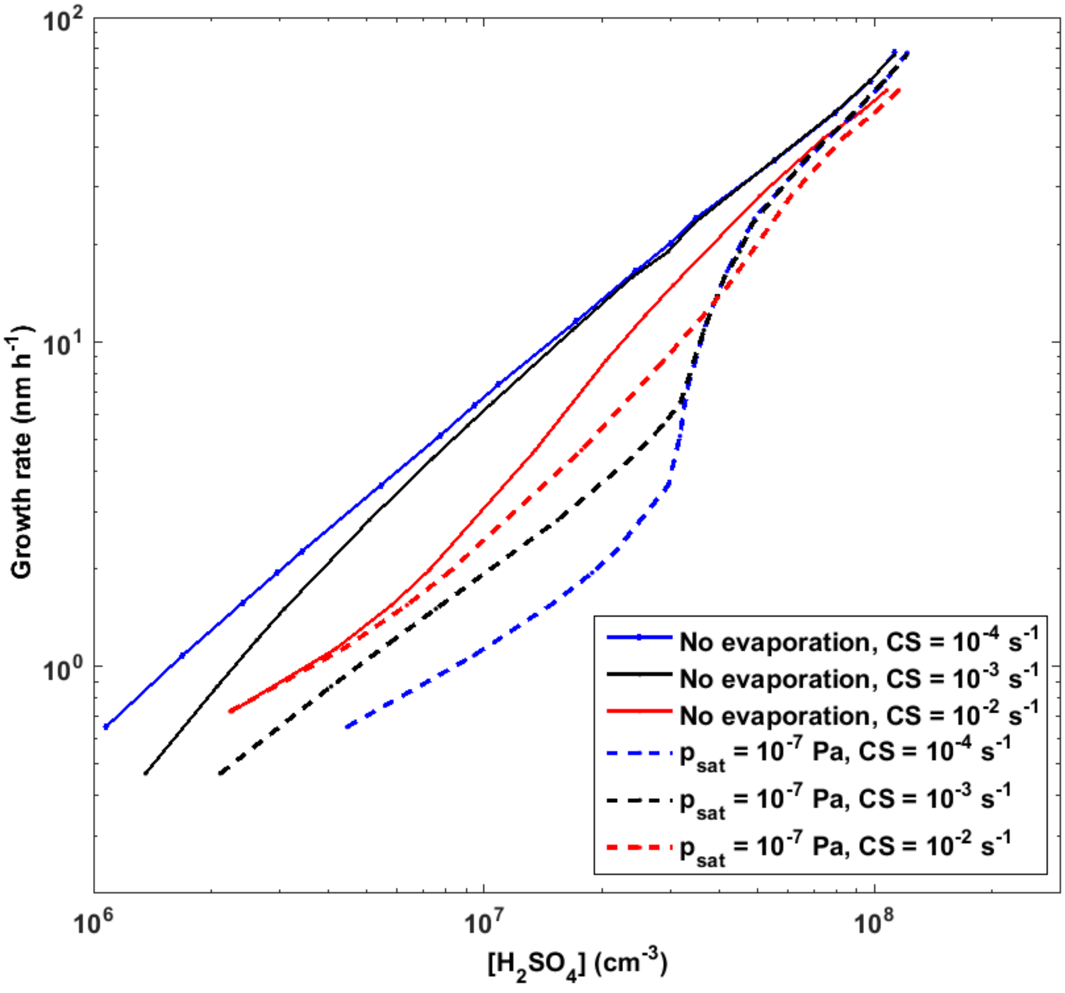

Supplementary Figure 7. Effect of condensation sink. The simulated appearance time growth rates as a function of sulphuric acid monomer concentration with no evaporation (solid lines) at different values of vapor condensation sink (CS; different colors) and for comparison a high evaporation rate at different values of condensation sink (dashed lines). When the condensation sink gets higher, the solid lines get closer to the dashed lines, indicating that the significance of evaporation and/or clustering to the growth rate gets smaller. The scavenging loss rate of clusters decreases with increasing cluster size, and the values CS in the legend correspond to the loss rate of the vapor monomer.

Supplementary references

1. Kürten, A. *et al.* Neutral molecular cluster formation of sulfuric acid–dimethylamine observed in real time under atmospheric conditions. *Proc. Natl.Acad. Sci. USA* **111**, 15019-15024 (2014).
